# 5a-Butyl-1,3,8,10-tetra­chloro-7,13-bis­(4-nitro­benzo­yl)-5a,6a,12a,12b-tetra­hydro-7*H*,13*H*-thieno[2,3-*b*:4,5-*b*′]bis­(1,4-benzoxazine)

**DOI:** 10.1107/S1600536812034149

**Published:** 2012-08-04

**Authors:** Kai Tang, Margaret E. Kastner

**Affiliations:** a Department of Chemistry, Bucknell University, Lewisburg, PA 17837, USA

## Abstract

The title compound, C_34_H_24_Cl_4_N_4_O_8_S, is a linear penta­cyclic system formed of two substituted benzoxazinyl groups fused to 2-*n*-butyl­tetra­hydro­thio­phene. The oxazine ring, which is fused to the *n*-butyl-substituted side of the thio­phene ring, is in a boat conformation. The other fused oxazine ring and the tetra­hydro­thiene ring are each in an envelope conformation. The bridgehead C atom α to both the S and N atoms forms the flap of each envelope. This results in a twist of the penta­cyclic system such that the dihedral angle between the terminal dichloro­benzene rings is 82.92 (8)°. In the crystal, inversion-related mol­ecules form a weakly hydrogen-bonded dimer, with two C—H⋯O inter­actions between an H atom on the oxazine ring and an amide O atom. Additionally, C—H⋯O inter­actions occur between an H atom on a screw-related nitro­benzene ring and an O atom on the nitro­benzene ring of one mol­ecule. One of the Cl atoms and the butyl group are disordered over two sets of sites with occupancy ratios of 0.94 (2):0.06 (2) and 0.624 (4):0.376 (4), respectively.

## Related literature

For the synthesis of the title compound, see: Heine *et al.* (1993 ▶). For the crystal structure of a related compound, see: Garbauskas *et al.* (1985 ▶).
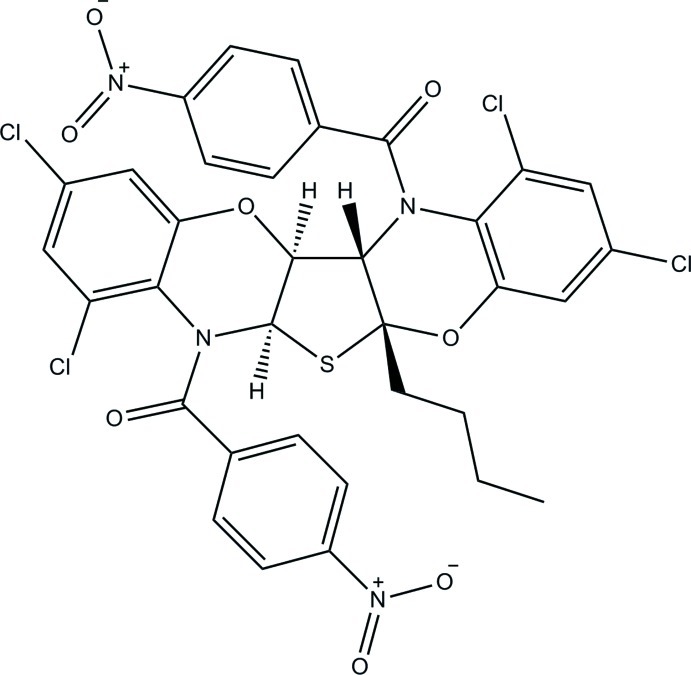


## Experimental

### 

#### Crystal data

C_34_H_24_Cl_4_N_4_O_8_S
*M* *_r_* = 790.44
Monoclinic, 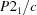

*a* = 12.855 (3) Å
*b* = 10.139 (1) Å
*c* = 27.845 (5) Å
β = 100.23 (2)°
*V* = 3571.5 (11) Å^3^
*Z* = 4
Mo *K*α radiation
μ = 0.45 mm^−1^
*T* = 293 K
0.5 × 0.2 × 0.1 mm

#### Data collection

Siemens R3m/V diffractometer
8579 measured reflections
8215 independent reflections
5039 reflections with *I* > 2σ(*I*)
*R*_int_ = 0.032
3 standard reflections every 97 reflections intensity decay: none

#### Refinement

*R*[*F* ^2^ > 2σ(*F* ^2^)] = 0.045
*wR*(*F* ^2^) = 0.123
*S* = 1.01
8215 reflections
476 parameters
6 restraints
H-atom parameters constrained
Δρ_max_ = 0.34 e Å^−3^
Δρ_min_ = −0.32 e Å^−3^

### 

Data collection: *XSCANS* (Siemens, 1996 ▶); cell refinement: *XSCANS*; data reduction: *XSCANS*; program(s) used to solve structure: *SHELXS97* (Sheldrick, 2008 ▶); program(s) used to refine structure: *SHELXL97* (Sheldrick, 2008 ▶); molecular graphics: *SHELXTL* (Sheldrick, 2008 ▶); software used to prepare material for publication: *SHELXTL*.

## Supplementary Material

Crystal structure: contains datablock(s) I, global. DOI: 10.1107/S1600536812034149/pv2572sup1.cif

Structure factors: contains datablock(s) I. DOI: 10.1107/S1600536812034149/pv2572Isup2.hkl

Additional supplementary materials:  crystallographic information; 3D view; checkCIF report

**Fig. 4.**
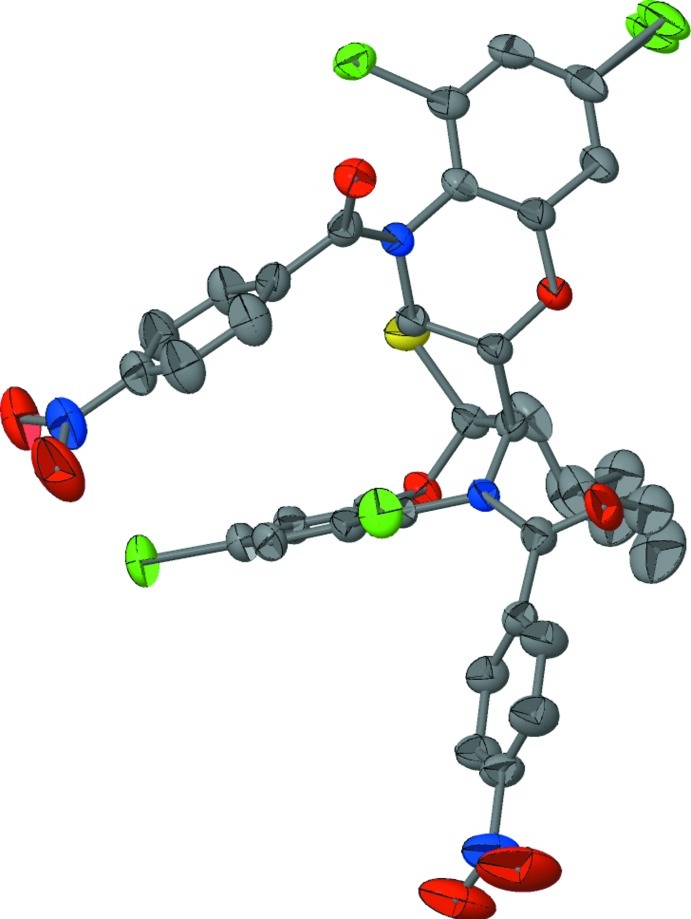
An enhanced figure of the molecule.

Enhanced figure: interactive version of Fig. 4

**Fig. 5.**
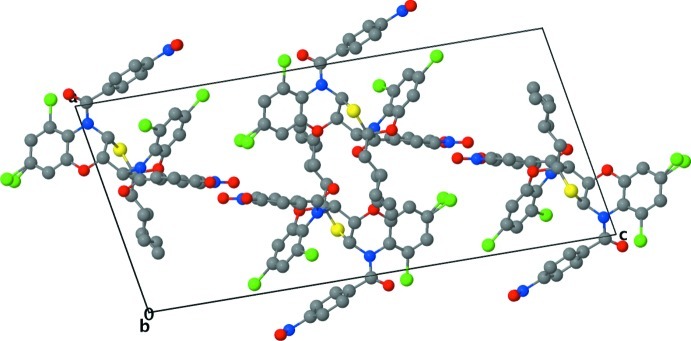
An enhanced figure of the unit cell.

Enhanced figure: interactive version of Fig. 5

## Figures and Tables

**Table 1:** Hydrogen-bond geometry (Å, °)

*D*—H⋯*A*	*D*—H	H⋯*A*	*D*⋯*A*	*D*—H⋯*A*
C2—H2⋯O2^i^	0.98	2.55	3.491 (3)	162
C14—H14⋯O3^ii^	0.93	2.41	3.298 (4)	160

Symmetry codes: (i) 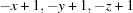; (ii) 
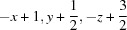
.

**Fig. 1.**
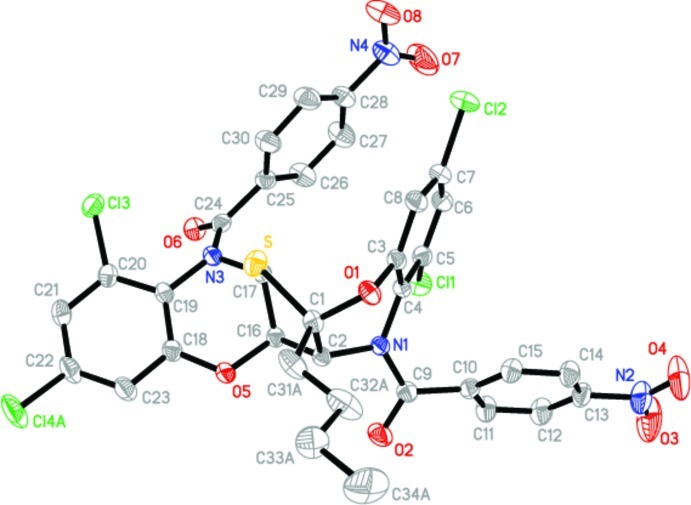
The molecular structure of the title compound, with atom labels and 30% probability displacement ellipsoids for non-H atoms. Cl4 and the n-butyl group are disordered and only one position is shown.

**Fig. 2.**
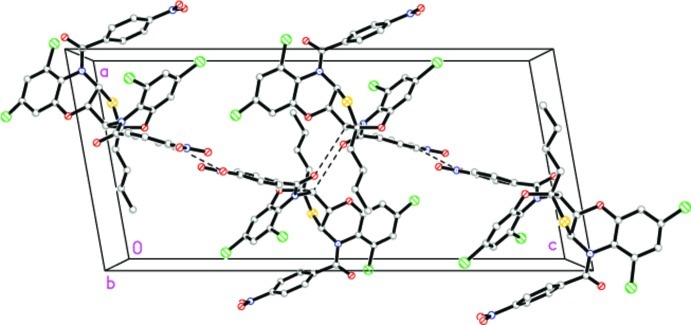
A view of the C—-H···O hydrogen bonds (dotted lines) in the crystal structure of the title compound. H atoms non-participating in hydrogen- bonding were omitted for clarity.

**Fig. 3.**
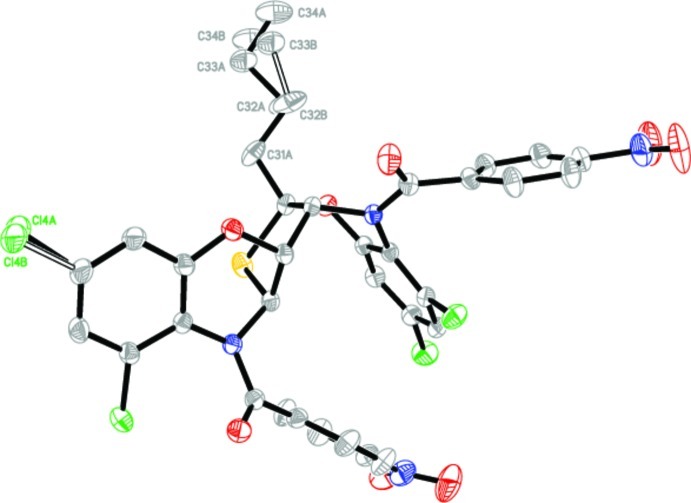
The molecular structure of the title compound with labels for the disordered atoms and 30% probability displacement ellipsoids for non-H atoms.

## supplementary crystallographic information

### Comment

The title compound was synthesized as part of a study of inverse electron-demand Diels-Alder reactions (Heine *et al.*, 1993).

The title molecule (Fig. 1), it is a linear pentacyclic system formed of two substituted benzoxazinyl groups fused to 2-n-butyltetrahydrothiophene. The oxazine ring (N1/O1/C1–C4) is in a boat conformation with O1 and N1 displaced by 0.536 (3) and 0.478 (3) Å, respectively, from the plane of C1–C4 atoms (r.m.s.d = 0.019 Å). In the dichlorobenzoxazinyl ring (O1/N2/Cl1–C8/Cl1/Cl2), the atoms O1/N1/C3–C8 lie in aplane (rmsd 0.0278 Å) while the atoms C1, C2, Cl1 and Cl2 lie 1.035 (3), 0.960 (3), 0.269 (2) and 0.014 (2) Å, respectively, out of this plane. In the other dichlorobenzoxazinyl ring (O5/N3/C16–C23/Cl3/Cl4), the atoms N3, O5, C16 and C18–C23, are coplanar (r.m.s.d = 0.0289 Å) while the atoms C17, Cl3, Cl4A and Cl4B are displaced from this plane by 0.553 (3), 0.170 (2), 0.52 (7) and 0.003 (8) Å, respectively. The dihedral angle between the terminal dichlorobenzene rings is 82.92 (8)*^o^*. The tetrahydrothiophene ring (S1/C1/C2/C16/C17) is in a C17- envelope conformation with C17 displaced by 0.630 (3)Å from the plane of the remaining ring atoms (r.m.s.d = 0.042 Å). The oxazine ring (N3/O5/C16–C19) is also in a C17-envelope conformation with C17 displaced by 0.572 (3) Å from the plane the other five atoms in the ring (r.m.s.d = 0.035 Å). The dihedral angles between the nitro groups and their associated benzene rings are: 5.9 (5)° for N/O3/O4 and 14.1 (3)° for N4/O7/O8.

In the crystal two inversion-related molecules form a weakly hydrogen-bonded dimer with C2—H2 ···O2 interactions (Table 1 and Fig. 2) Additionally, C14—H14···O3 hydrogen bonding interactions form between an oxygen on the nitrobenzene ring of one molecule and a hydrogen on a screw related nitrobenzene ring.

### Experimental

The title compound was synthesized by following a reported procedure (Heine *et al.*, (1993). The crystals suitable for X-ray crystallographic analysis were grown from a solution of acetonitrile by slow evaporation at room temperature.

### Refinement

The H atoms were positioned geometrically and refined using a riding model, with C—H = 0.93, 0.96, 0.97 and 0.98 Å, for aryl, methyl, methylene and methyne H-atoms, respectively. The *U*_iso_(H) were allowed at 1.5*U*_eq_(C methyl) or 1.2*U*_eq_(C non-methyl).

The *n*-butyl group was disordered [ratio: 0.624 (4):0.376 (4)] and the bond distances were constrained to chemically acceptable values. A chlorine atom, Cl4, was also disordered with a population ratio of 0.94 (2):0.06 (2).

### Crystal data

**Table:**

C_34_H_24_Cl_4_N_4_O_8_S	*F*(000) = 1616
*M**_r_* = 790.44	*D*_x_ = 1.470 Mg m^−^^3^
Monoclinic, *P*2_1_/*c*	Mo *K*α radiation, λ = 0.71073 Å
Hall symbol: -P 2ybc	Cell parameters from 20 reflections
*a* = 12.855 (3) Å	θ = 10–12.5°
*b* = 10.139 (1) Å	µ = 0.45 mm^−^^1^
*c* = 27.845 (5) Å	*T* = 293 K
β = 100.23 (2)°	Needle, colorless
*V* = 3571.5 (11) Å^3^	0.5 × 0.2 × 0.1 mm
*Z* = 4	

### Data collection

**Table:**

Siemens R3m/V diffractometer	*R*_int_ = 0.032
Radiation source: fine-focus sealed tube	θ_max_ = 27.6°, θ_min_ = 1.6°
Graphite monochromator	*h* = 0→16
θ 2 θ scans	*k* = 0→13
8579 measured reflections	*l* = −36→35
8215 independent reflections	3 standard reflections every 97 reflections
5039 reflections with *I* > 2σ(*I*)	intensity decay: none

### Refinement

**Table:**

Refinement on *F*^2^	Primary atom site location: structure-invariant direct methods
Least-squares matrix: full	Secondary atom site location: difference Fourier map
*R*[*F*^2^ > 2σ(*F*^2^)] = 0.045	Hydrogen site location: inferred from neighbouring sites
*wR*(*F*^2^) = 0.123	H-atom parameters constrained
*S* = 1.01	*w* = 1/[σ^2^(*F*_o_^2^) + (0.0624*P*)^2^] where *P* = (*F*_o_^2^ + 2*F*_c_^2^)/3
8215 reflections	(Δ/σ)_max_ = 0.017
476 parameters	Δρ_max_ = 0.34 e Å^−^^3^
6 restraints	Δρ_min_ = −0.32 e Å^−^^3^

### Special details

**Table:**

Geometry. All e.s.d.'s (except the e.s.d. in the dihedral angle between two l.s. planes) are estimated using the full covariance matrix. The cell e.s.d.'s are taken into account individually in the estimation of e.s.d.'s in distances, angles and torsion angles; correlations between e.s.d.'s in cell parameters are only used when they are defined by crystal symmetry. An approximate (isotropic) treatment of cell e.s.d.'s is used for estimating e.s.d.'s involving l.s. planes.
Refinement. Refinement of *F*^2^ against ALL reflections. The weighted *R*-factor *wR* and goodness of fit *S* are based on *F*^2^, conventional *R*-factors *R* are based on *F*, with *F* set to zero for negative *F*^2^. The threshold expression of *F*^2^ > σ(*F*^2^) is used only for calculating *R*-factors(gt) *etc*. and is not relevant to the choice of reflections for refinement. *R*-factors based on *F*^2^ are statistically about twice as large as those based on *F*, and *R*- factors based on ALL data will be even larger.

### Fractional atomic coordinates and isotropic or equivalent isotropic displacement parameters (Å^2^)

**Table:**

	*x*	*y*	*z*	*U*_iso_*/*U*_eq_	Occ. (<1)
Cl1	0.86026 (6)	0.33223 (7)	0.62869 (3)	0.05638 (19)	
Cl2	0.95304 (6)	0.70359 (9)	0.76649 (3)	0.0707 (2)	
Cl3	1.04827 (5)	0.89267 (7)	0.45592 (2)	0.05211 (18)	
Cl4A	0.704 (3)	0.903 (6)	0.3231 (17)	0.1013 (10)	0.06 (2)
Cl4B	0.7177 (2)	0.8654 (5)	0.31026 (17)	0.1013 (10)	0.94 (2)
S	0.76688 (6)	0.84957 (6)	0.55950 (2)	0.04857 (18)	
O1	0.64969 (13)	0.75499 (16)	0.62293 (5)	0.0394 (4)	
O2	0.57552 (17)	0.37288 (18)	0.53786 (6)	0.0611 (5)	
O3	0.5516 (3)	−0.0704 (3)	0.71927 (11)	0.1308 (13)	
O4	0.5328 (3)	0.0893 (3)	0.76668 (10)	0.1139 (11)	
O5	0.70106 (13)	0.65736 (18)	0.47193 (5)	0.0461 (4)	
O6	1.06030 (14)	0.62308 (17)	0.49661 (6)	0.0481 (4)	
O7	1.2038 (3)	0.5453 (3)	0.74913 (9)	0.1113 (10)	
O8	1.2033 (2)	0.7573 (3)	0.75138 (8)	0.0896 (8)	
N1	0.67147 (14)	0.51822 (18)	0.58817 (6)	0.0334 (4)	
N2	0.5500 (2)	0.0458 (3)	0.72858 (11)	0.0791 (8)	
N3	0.91180 (15)	0.72470 (19)	0.51363 (6)	0.0373 (4)	
N4	1.1870 (2)	0.6530 (3)	0.72987 (10)	0.0715 (7)	
C1	0.65006 (19)	0.7586 (2)	0.57105 (8)	0.0369 (5)	
C2	0.65631 (18)	0.6171 (2)	0.54903 (7)	0.0325 (5)	
H2	0.5894	0.5986	0.5271	0.039*	
C3	0.72924 (18)	0.6790 (2)	0.64898 (8)	0.0357 (5)	
C4	0.74216 (17)	0.5532 (2)	0.63131 (8)	0.0335 (5)	
C5	0.82759 (19)	0.4781 (2)	0.65429 (8)	0.0392 (5)	
C6	0.8903 (2)	0.5241 (3)	0.69675 (9)	0.0460 (6)	
H6	0.9452	0.4726	0.7132	0.055*	
C7	0.8704 (2)	0.6469 (3)	0.71427 (8)	0.0471 (6)	
C8	0.7918 (2)	0.7280 (3)	0.69062 (8)	0.0441 (6)	
H8	0.7813	0.8121	0.7022	0.053*	
C9	0.6174 (2)	0.4013 (2)	0.57913 (8)	0.0394 (5)	
C10	0.6058 (2)	0.3136 (2)	0.62103 (9)	0.0403 (6)	
C11	0.6156 (2)	0.1783 (2)	0.61429 (10)	0.0538 (7)	
H11	0.6343	0.1471	0.5856	0.065*	
C12	0.5980 (2)	0.0908 (3)	0.64951 (11)	0.0592 (8)	
H12	0.6059	0.0005	0.6455	0.071*	
C13	0.5685 (2)	0.1405 (3)	0.69056 (10)	0.0509 (7)	
C14	0.5575 (2)	0.2728 (3)	0.69851 (10)	0.0533 (7)	
H14	0.5375	0.3030	0.7271	0.064*	
C15	0.5767 (2)	0.3602 (2)	0.66308 (9)	0.0453 (6)	
H15	0.5699	0.4505	0.6676	0.054*	
C16	0.74536 (17)	0.6109 (2)	0.51966 (7)	0.0335 (5)	
H16	0.7711	0.5202	0.5182	0.040*	
C17	0.83346 (18)	0.7009 (2)	0.54434 (8)	0.0351 (5)	
H17	0.8684	0.6591	0.5747	0.042*	
C18	0.76444 (19)	0.7222 (2)	0.44547 (8)	0.0403 (6)	
C19	0.86932 (19)	0.7570 (2)	0.46436 (8)	0.0368 (5)	
C20	0.92359 (19)	0.8300 (2)	0.43419 (9)	0.0410 (6)	
C21	0.8790 (2)	0.8600 (3)	0.38661 (9)	0.0516 (7)	
H21	0.9178	0.9044	0.3665	0.062*	
C22	0.7756 (2)	0.8230 (3)	0.36943 (9)	0.0587 (8)	
C23	0.7176 (2)	0.7562 (3)	0.39837 (9)	0.0538 (7)	
H23	0.6476	0.7340	0.3865	0.065*	
C24	1.01125 (19)	0.6649 (2)	0.52636 (9)	0.0392 (5)	
C25	1.05379 (19)	0.6615 (2)	0.58040 (9)	0.0422 (6)	
C26	1.0972 (2)	0.5471 (3)	0.60153 (10)	0.0582 (8)	
H26	1.0970	0.4715	0.5826	0.070*	
C27	1.1411 (2)	0.5429 (3)	0.65061 (11)	0.0658 (8)	
H27	1.1695	0.4651	0.6650	0.079*	
C28	1.1417 (2)	0.6559 (3)	0.67727 (9)	0.0535 (7)	
C29	1.0998 (2)	0.7720 (3)	0.65746 (10)	0.0618 (8)	
H29	1.1024	0.8479	0.6764	0.074*	
C30	1.0537 (2)	0.7742 (3)	0.60879 (10)	0.0584 (8)	
H30	1.0225	0.8512	0.5950	0.070*	
C31A	0.5497 (2)	0.8317 (3)	0.54905 (9)	0.0552 (7)	
H31A	0.5615	0.9252	0.5549	0.066*	0.624 (4)
H31B	0.5374	0.8183	0.5140	0.066*	0.624 (4)
C32A	0.4516 (5)	0.7932 (9)	0.5675 (4)	0.074 (2)	0.624 (4)
H32A	0.4621	0.8011	0.6028	0.089*	0.624 (4)
H32B	0.4320	0.7030	0.5585	0.089*	0.624 (4)
C33A	0.3653 (4)	0.8902 (6)	0.5431 (2)	0.0687 (13)	0.624 (4)
H33A	0.3861	0.9804	0.5515	0.082*	0.624 (4)
H33B	0.3544	0.8810	0.5079	0.082*	0.624 (4)
C34A	0.2662 (4)	0.8563 (8)	0.5617 (3)	0.104 (2)	0.624 (4)
H34A	0.2399	0.7728	0.5485	0.156*	0.624 (4)
H34B	0.2139	0.9232	0.5518	0.156*	0.624 (4)
H34C	0.2811	0.8511	0.5966	0.156*	0.624 (4)
H31C	0.5451	0.8378	0.5140	0.066*	0.376 (4)
H31D	0.5522	0.9205	0.5621	0.066*	0.376 (4)
C32B	0.4516 (7)	0.7606 (17)	0.5604 (7)	0.074 (2)	0.376 (4)
H32C	0.4613	0.7552	0.5957	0.089*	0.376 (4)
H32D	0.4561	0.6709	0.5489	0.089*	0.376 (4)
C33B	0.3355 (7)	0.8037 (9)	0.5432 (4)	0.0687 (13)	0.376 (4)
H33C	0.3209	0.8099	0.5079	0.082*	0.376 (4)
H33D	0.2885	0.7381	0.5530	0.082*	0.376 (4)
C34B	0.3156 (9)	0.9355 (11)	0.5651 (5)	0.104 (2)	0.376 (4)
H34D	0.3310	0.9295	0.6000	0.156*	0.376 (4)
H34E	0.2429	0.9598	0.5548	0.156*	0.376 (4)
H34F	0.3603	1.0010	0.5543	0.156*	0.376 (4)

### Atomic displacement parameters (Å^2^)

**Table:**

	*U*^11^	*U*^22^	*U*^33^	*U*^12^	*U*^13^	*U*^23^
Cl1	0.0574 (4)	0.0453 (4)	0.0640 (4)	0.0154 (3)	0.0044 (3)	−0.0023 (3)
Cl2	0.0673 (5)	0.0899 (6)	0.0467 (4)	−0.0176 (4)	−0.0120 (3)	−0.0106 (4)
Cl3	0.0555 (4)	0.0480 (4)	0.0555 (4)	−0.0114 (3)	0.0173 (3)	0.0002 (3)
Cl4A	0.0683 (7)	0.195 (2)	0.0404 (11)	0.0100 (9)	0.0105 (7)	0.0446 (13)
Cl4B	0.0683 (7)	0.195 (2)	0.0404 (11)	0.0100 (9)	0.0105 (7)	0.0446 (13)
S	0.0659 (5)	0.0321 (3)	0.0518 (4)	−0.0050 (3)	0.0216 (3)	−0.0053 (3)
O1	0.0462 (9)	0.0421 (9)	0.0305 (8)	0.0102 (8)	0.0081 (7)	−0.0036 (7)
O2	0.0892 (15)	0.0464 (11)	0.0408 (10)	−0.0161 (10)	−0.0071 (10)	−0.0045 (8)
O3	0.229 (4)	0.0590 (17)	0.125 (2)	−0.021 (2)	0.085 (3)	0.0261 (16)
O4	0.194 (3)	0.092 (2)	0.0727 (16)	0.003 (2)	0.0686 (19)	0.0203 (15)
O5	0.0429 (10)	0.0654 (12)	0.0296 (8)	−0.0093 (9)	0.0054 (7)	0.0029 (8)
O6	0.0498 (11)	0.0483 (10)	0.0480 (10)	0.0046 (9)	0.0132 (8)	−0.0026 (8)
O7	0.147 (3)	0.102 (2)	0.0679 (16)	0.0114 (19)	−0.0277 (16)	0.0222 (15)
O8	0.0913 (18)	0.106 (2)	0.0600 (14)	0.0006 (15)	−0.0163 (12)	−0.0223 (14)
N1	0.0385 (11)	0.0317 (10)	0.0291 (9)	−0.0023 (8)	0.0038 (8)	−0.0002 (8)
N2	0.102 (2)	0.069 (2)	0.0735 (19)	−0.0069 (17)	0.0343 (17)	0.0208 (16)
N3	0.0373 (11)	0.0438 (11)	0.0303 (10)	−0.0007 (9)	0.0047 (8)	0.0020 (8)
N4	0.0691 (18)	0.087 (2)	0.0508 (15)	0.0045 (16)	−0.0092 (13)	0.0002 (15)
C1	0.0467 (14)	0.0328 (12)	0.0320 (11)	0.0063 (11)	0.0094 (10)	−0.0013 (9)
C2	0.0356 (12)	0.0309 (11)	0.0302 (11)	0.0005 (10)	0.0041 (9)	−0.0007 (9)
C3	0.0401 (13)	0.0385 (13)	0.0291 (11)	−0.0004 (10)	0.0082 (9)	−0.0006 (9)
C4	0.0379 (13)	0.0339 (12)	0.0288 (11)	−0.0028 (10)	0.0059 (9)	0.0007 (9)
C5	0.0409 (14)	0.0358 (13)	0.0403 (13)	0.0009 (11)	0.0061 (10)	0.0049 (10)
C6	0.0436 (15)	0.0502 (15)	0.0410 (14)	−0.0003 (12)	−0.0018 (11)	0.0096 (12)
C7	0.0487 (15)	0.0562 (16)	0.0334 (12)	−0.0095 (13)	−0.0006 (11)	−0.0007 (11)
C8	0.0529 (15)	0.0437 (14)	0.0354 (13)	−0.0046 (12)	0.0068 (11)	−0.0051 (11)
C9	0.0471 (14)	0.0333 (12)	0.0376 (13)	−0.0004 (11)	0.0067 (11)	−0.0038 (10)
C10	0.0456 (14)	0.0353 (13)	0.0399 (13)	−0.0074 (11)	0.0078 (11)	−0.0019 (10)
C11	0.083 (2)	0.0342 (14)	0.0486 (15)	−0.0082 (13)	0.0236 (14)	−0.0053 (11)
C12	0.085 (2)	0.0327 (14)	0.0626 (18)	−0.0065 (14)	0.0212 (16)	0.0009 (13)
C13	0.0582 (17)	0.0443 (15)	0.0527 (16)	−0.0083 (13)	0.0166 (13)	0.0093 (12)
C14	0.0617 (18)	0.0567 (17)	0.0469 (15)	−0.0014 (14)	0.0245 (13)	−0.0017 (13)
C15	0.0530 (16)	0.0354 (13)	0.0503 (15)	−0.0003 (12)	0.0166 (12)	−0.0025 (11)
C16	0.0405 (13)	0.0317 (11)	0.0294 (11)	0.0003 (10)	0.0090 (9)	−0.0007 (9)
C17	0.0409 (13)	0.0343 (12)	0.0305 (11)	−0.0021 (10)	0.0072 (10)	0.0004 (9)
C18	0.0436 (14)	0.0474 (14)	0.0318 (12)	0.0003 (11)	0.0120 (10)	−0.0017 (10)
C19	0.0425 (13)	0.0384 (13)	0.0302 (11)	0.0037 (11)	0.0084 (10)	−0.0001 (10)
C20	0.0459 (14)	0.0395 (13)	0.0409 (13)	0.0047 (11)	0.0168 (11)	0.0014 (11)
C21	0.0589 (18)	0.0593 (17)	0.0406 (14)	0.0050 (14)	0.0196 (12)	0.0096 (12)
C22	0.0577 (18)	0.087 (2)	0.0319 (13)	0.0132 (16)	0.0105 (12)	0.0116 (14)
C23	0.0450 (15)	0.081 (2)	0.0350 (13)	0.0040 (14)	0.0062 (11)	0.0062 (13)
C24	0.0400 (13)	0.0312 (12)	0.0454 (13)	−0.0050 (11)	0.0042 (11)	0.0010 (10)
C25	0.0373 (13)	0.0427 (14)	0.0447 (13)	−0.0008 (11)	0.0022 (11)	0.0000 (11)
C26	0.0675 (19)	0.0477 (16)	0.0551 (17)	0.0168 (14)	−0.0011 (14)	−0.0082 (13)
C27	0.073 (2)	0.0572 (18)	0.0605 (18)	0.0225 (16)	−0.0067 (16)	0.0054 (15)
C28	0.0487 (16)	0.0618 (18)	0.0453 (15)	0.0036 (14)	−0.0040 (12)	0.0029 (13)
C29	0.077 (2)	0.0492 (17)	0.0542 (17)	−0.0015 (15)	−0.0024 (15)	−0.0121 (14)
C30	0.074 (2)	0.0413 (15)	0.0522 (16)	0.0025 (14)	−0.0095 (14)	−0.0007 (13)
C31A	0.0674 (19)	0.0580 (17)	0.0371 (13)	0.0326 (15)	0.0008 (12)	−0.0029 (12)
C32A	0.0482 (18)	0.132 (6)	0.044 (4)	0.040 (2)	0.0079 (16)	−0.020 (4)
C33A	0.051 (3)	0.076 (4)	0.081 (3)	0.016 (3)	0.016 (2)	0.003 (3)
C34A	0.047 (4)	0.133 (7)	0.134 (5)	0.019 (3)	0.023 (4)	−0.014 (5)
C32B	0.0482 (18)	0.132 (6)	0.044 (4)	0.040 (2)	0.0079 (16)	−0.020 (4)
C33B	0.051 (3)	0.076 (4)	0.081 (3)	0.016 (3)	0.016 (2)	0.003 (3)
C34B	0.047 (4)	0.133 (7)	0.134 (5)	0.019 (3)	0.023 (4)	−0.014 (5)

### Geometric parameters (Å, º)

**Table:**

Cl1—C5	1.726 (2)	C14—H14	0.9300
Cl2—C7	1.739 (2)	C15—H15	0.9300
Cl3—C20	1.730 (3)	C16—C17	1.520 (3)
Cl4A—C22	1.66 (3)	C16—H16	0.9800
Cl4B—C22	1.737 (4)	C17—H17	0.9800
S—C17	1.820 (2)	C18—C23	1.386 (3)
S—C1	1.839 (3)	C18—C19	1.402 (3)
O1—C3	1.379 (3)	C19—C20	1.396 (3)
O1—C1	1.446 (3)	C20—C21	1.381 (3)
O2—C9	1.214 (3)	C21—C22	1.383 (4)
O3—N2	1.207 (4)	C21—H21	0.9300
O4—N2	1.205 (3)	C22—C23	1.370 (4)
O5—C18	1.361 (3)	C23—H23	0.9300
O5—C16	1.430 (3)	C24—C25	1.507 (3)
O6—C24	1.204 (3)	C25—C26	1.373 (3)
O7—N4	1.219 (4)	C25—C30	1.389 (3)
O8—N4	1.214 (3)	C26—C27	1.384 (4)
N1—C9	1.375 (3)	C26—H26	0.9300
N1—C4	1.417 (3)	C27—C28	1.365 (4)
N1—C2	1.468 (3)	C27—H27	0.9300
N2—C13	1.479 (3)	C28—C29	1.369 (4)
N3—C24	1.402 (3)	C29—C30	1.379 (4)
N3—C19	1.422 (3)	C29—H29	0.9300
N3—C17	1.452 (3)	C30—H30	0.9300
N4—C28	1.477 (4)	C31A—C32A	1.495 (7)
C1—C31A	1.519 (3)	C31A—H31A	0.9700
C1—C2	1.568 (3)	C31A—H31B	0.9700
C2—C16	1.522 (3)	C32A—C33A	1.546 (7)
C2—H2	0.9800	C32A—H32A	0.9700
C3—C8	1.381 (3)	C32A—H32B	0.9700
C3—C4	1.387 (3)	C33A—C34A	1.498 (7)
C4—C5	1.395 (3)	C33A—H33A	0.9700
C5—C6	1.388 (3)	C33A—H33B	0.9700
C6—C7	1.378 (4)	C34A—H34A	0.9600
C6—H6	0.9300	C34A—H34B	0.9600
C7—C8	1.376 (4)	C34A—H34C	0.9600
C8—H8	0.9300	C32B—C33B	1.547 (9)
C9—C10	1.495 (3)	C32B—H32C	0.9700
C10—C15	1.375 (3)	C32B—H32D	0.9700
C10—C11	1.393 (3)	C33B—C34B	1.508 (9)
C11—C12	1.371 (4)	C33B—H33C	0.9700
C11—H11	0.9300	C33B—H33D	0.9700
C12—C13	1.363 (4)	C34B—H34D	0.9600
C12—H12	0.9300	C34B—H34E	0.9600
C13—C14	1.371 (4)	C34B—H34F	0.9600
C14—C15	1.381 (3)		
			
C17—S—C1	93.28 (10)	C16—C17—H17	108.7
C3—O1—C1	113.94 (16)	S—C17—H17	108.7
C18—O5—C16	118.98 (17)	O5—C18—C23	115.5 (2)
C9—N1—C4	126.79 (18)	O5—C18—C19	123.1 (2)
C9—N1—C2	117.17 (18)	C23—C18—C19	121.3 (2)
C4—N1—C2	116.04 (17)	C20—C19—C18	117.2 (2)
O4—N2—O3	124.0 (3)	C20—C19—N3	123.7 (2)
O4—N2—C13	118.1 (3)	C18—C19—N3	118.9 (2)
O3—N2—C13	118.0 (3)	C21—C20—C19	121.8 (2)
C24—N3—C19	121.34 (19)	C21—C20—Cl3	117.11 (19)
C24—N3—C17	118.40 (19)	C19—C20—Cl3	121.00 (18)
C19—N3—C17	114.75 (18)	C20—C21—C22	118.8 (2)
O8—N4—O7	124.1 (3)	C20—C21—H21	120.6
O8—N4—C28	118.3 (3)	C22—C21—H21	120.6
O7—N4—C28	117.5 (3)	C23—C22—C21	121.4 (2)
O1—C1—C31A	105.18 (18)	C23—C22—Cl4A	114.3 (13)
O1—C1—C2	112.17 (17)	C21—C22—Cl4A	120.4 (11)
C31A—C1—C2	112.8 (2)	C23—C22—Cl4B	119.6 (2)
O1—C1—S	109.56 (15)	C21—C22—Cl4B	119.0 (2)
C31A—C1—S	110.25 (18)	C22—C23—C18	119.3 (3)
C2—C1—S	106.90 (15)	C22—C23—H23	120.4
N1—C2—C16	110.95 (18)	C18—C23—H23	120.4
N1—C2—C1	110.10 (16)	O6—C24—N3	122.9 (2)
C16—C2—C1	110.10 (18)	O6—C24—C25	122.5 (2)
N1—C2—H2	108.5	N3—C24—C25	114.5 (2)
C16—C2—H2	108.5	C26—C25—C30	119.5 (2)
C1—C2—H2	108.5	C26—C25—C24	119.6 (2)
O1—C3—C8	120.5 (2)	C30—C25—C24	120.9 (2)
O1—C3—C4	116.83 (19)	C25—C26—C27	120.9 (3)
C8—C3—C4	122.7 (2)	C25—C26—H26	119.6
C3—C4—C5	118.2 (2)	C27—C26—H26	119.6
C3—C4—N1	115.59 (19)	C28—C27—C26	118.2 (3)
C5—C4—N1	125.9 (2)	C28—C27—H27	120.9
C6—C5—C4	119.9 (2)	C26—C27—H27	120.9
C6—C5—Cl1	119.49 (19)	C27—C28—C29	122.6 (3)
C4—C5—Cl1	120.52 (18)	C27—C28—N4	119.0 (3)
C7—C6—C5	119.2 (2)	C29—C28—N4	118.3 (3)
C7—C6—H6	120.4	C28—C29—C30	118.6 (3)
C5—C6—H6	120.4	C28—C29—H29	120.7
C8—C7—C6	122.5 (2)	C30—C29—H29	120.7
C8—C7—Cl2	119.4 (2)	C29—C30—C25	120.2 (3)
C6—C7—Cl2	118.1 (2)	C29—C30—H30	119.9
C7—C8—C3	117.1 (2)	C25—C30—H30	119.9
C7—C8—H8	121.4	C32A—C31A—C1	116.2 (4)
C3—C8—H8	121.4	C32A—C31A—H31A	108.2
O2—C9—N1	120.2 (2)	C1—C31A—H31A	108.2
O2—C9—C10	120.6 (2)	C32A—C31A—H31B	108.2
N1—C9—C10	119.1 (2)	C1—C31A—H31B	108.2
C15—C10—C11	119.9 (2)	H31A—C31A—H31B	107.4
C15—C10—C9	122.7 (2)	C31A—C32A—C33A	105.4 (5)
C11—C10—C9	117.2 (2)	C31A—C32A—H32A	110.7
C12—C11—C10	120.7 (2)	C33A—C32A—H32A	110.7
C12—C11—H11	119.6	C31A—C32A—H32B	110.7
C10—C11—H11	119.6	C33A—C32A—H32B	110.7
C13—C12—C11	117.8 (3)	H32A—C32A—H32B	108.8
C13—C12—H12	121.1	C34A—C33A—C32A	106.9 (5)
C11—C12—H12	121.1	C34A—C33A—H33A	110.3
C12—C13—C14	123.3 (2)	C32A—C33A—H33A	110.4
C12—C13—N2	117.7 (3)	C34A—C33A—H33B	110.4
C14—C13—N2	119.0 (3)	C32A—C33A—H33B	110.4
C13—C14—C15	118.4 (2)	H33A—C33A—H33B	108.6
C13—C14—H14	120.8	C33B—C32B—H32C	105.8
C15—C14—H14	120.8	C33B—C32B—H32D	105.8
C10—C15—C14	119.8 (2)	H32C—C32B—H32D	106.2
C10—C15—H15	120.1	C34B—C33B—C32B	110.4 (11)
C14—C15—H15	120.1	C34B—C33B—H33C	109.6
O5—C16—C17	111.36 (18)	C32B—C33B—H33C	109.6
O5—C16—C2	105.82 (17)	C34B—C33B—H33D	109.6
C17—C16—C2	107.66 (17)	C32B—C33B—H33D	109.6
O5—C16—H16	110.6	H33C—C33B—H33D	108.1
C17—C16—H16	110.6	C33B—C34B—H34D	109.5
C2—C16—H16	110.6	C33B—C34B—H34E	109.5
N3—C17—C16	111.85 (17)	H34D—C34B—H34E	109.5
N3—C17—S	113.76 (16)	C33B—C34B—H34F	109.5
C16—C17—S	105.00 (15)	H34D—C34B—H34F	109.5
N3—C17—H17	108.7	H34E—C34B—H34F	109.5
			
C3—O1—C1—C31A	173.0 (2)	N1—C2—C16—C17	−88.4 (2)
C3—O1—C1—C2	50.0 (2)	C1—C2—C16—C17	33.7 (2)
C3—O1—C1—S	−68.5 (2)	C24—N3—C17—C16	−107.8 (2)
C17—S—C1—O1	107.13 (15)	C19—N3—C17—C16	46.4 (3)
C17—S—C1—C31A	−137.58 (17)	C24—N3—C17—S	133.40 (18)
C17—S—C1—C2	−14.64 (15)	C19—N3—C17—S	−72.4 (2)
C9—N1—C2—C16	−97.4 (2)	O5—C16—C17—N3	−51.8 (2)
C4—N1—C2—C16	81.7 (2)	C2—C16—C17—N3	−167.39 (18)
C9—N1—C2—C1	140.5 (2)	O5—C16—C17—S	72.02 (19)
C4—N1—C2—C1	−40.5 (2)	C2—C16—C17—S	−43.55 (19)
O1—C1—C2—N1	−6.0 (3)	C1—S—C17—N3	156.25 (16)
C31A—C1—C2—N1	−124.5 (2)	C1—S—C17—C16	33.63 (15)
S—C1—C2—N1	114.15 (17)	C16—O5—C18—C23	175.8 (2)
O1—C1—C2—C16	−128.59 (19)	C16—O5—C18—C19	−6.2 (3)
C31A—C1—C2—C16	112.9 (2)	O5—C18—C19—C20	−176.4 (2)
S—C1—C2—C16	−8.5 (2)	C23—C18—C19—C20	1.6 (4)
C1—O1—C3—C8	131.2 (2)	O5—C18—C19—N3	−0.7 (4)
C1—O1—C3—C4	−48.9 (3)	C23—C18—C19—N3	177.2 (2)
O1—C3—C4—C5	174.5 (2)	C24—N3—C19—C20	−51.9 (3)
C8—C3—C4—C5	−5.6 (3)	C17—N3—C19—C20	154.7 (2)
O1—C3—C4—N1	0.1 (3)	C24—N3—C19—C18	132.7 (2)
C8—C3—C4—N1	−180.0 (2)	C17—N3—C19—C18	−20.7 (3)
C9—N1—C4—C3	−134.9 (2)	C18—C19—C20—C21	−4.1 (4)
C2—N1—C4—C3	46.1 (3)	N3—C19—C20—C21	−179.5 (2)
C9—N1—C4—C5	51.2 (3)	C18—C19—C20—Cl3	173.25 (18)
C2—N1—C4—C5	−127.8 (2)	N3—C19—C20—Cl3	−2.2 (3)
C3—C4—C5—C6	6.7 (3)	C19—C20—C21—C22	3.8 (4)
N1—C4—C5—C6	−179.6 (2)	Cl3—C20—C21—C22	−173.7 (2)
C3—C4—C5—Cl1	−169.92 (17)	C20—C21—C22—C23	−0.8 (4)
N1—C4—C5—Cl1	3.8 (3)	C20—C21—C22—Cl4A	156 (3)
C4—C5—C6—C7	−3.3 (4)	C20—C21—C22—Cl4B	177.8 (3)
Cl1—C5—C6—C7	173.38 (19)	C21—C22—C23—C18	−1.6 (5)
C5—C6—C7—C8	−1.6 (4)	Cl4A—C22—C23—C18	−160 (3)
C5—C6—C7—Cl2	−178.06 (19)	Cl4B—C22—C23—C18	179.7 (3)
C6—C7—C8—C3	2.8 (4)	O5—C18—C23—C22	179.3 (3)
Cl2—C7—C8—C3	179.19 (18)	C19—C18—C23—C22	1.2 (4)
O1—C3—C8—C7	−179.2 (2)	C19—N3—C24—O6	−9.3 (3)
C4—C3—C8—C7	0.9 (4)	C17—N3—C24—O6	143.2 (2)
C4—N1—C9—O2	−165.7 (2)	C19—N3—C24—C25	168.6 (2)
C2—N1—C9—O2	13.3 (3)	C17—N3—C24—C25	−39.0 (3)
C4—N1—C9—C10	17.7 (3)	O6—C24—C25—C26	−48.0 (4)
C2—N1—C9—C10	−163.4 (2)	N3—C24—C25—C26	134.1 (3)
O2—C9—C10—C15	−130.6 (3)	O6—C24—C25—C30	129.3 (3)
N1—C9—C10—C15	46.1 (4)	N3—C24—C25—C30	−48.5 (3)
O2—C9—C10—C11	43.3 (4)	C30—C25—C26—C27	−0.3 (4)
N1—C9—C10—C11	−140.1 (3)	C24—C25—C26—C27	177.1 (3)
C15—C10—C11—C12	−0.9 (4)	C25—C26—C27—C28	−1.0 (5)
C9—C10—C11—C12	−175.0 (3)	C26—C27—C28—C29	0.6 (5)
C10—C11—C12—C13	1.4 (5)	C26—C27—C28—N4	179.2 (3)
C11—C12—C13—C14	−1.1 (5)	O8—N4—C28—C27	167.9 (3)
C11—C12—C13—N2	−179.8 (3)	O7—N4—C28—C27	−13.4 (5)
O4—N2—C13—C12	173.8 (3)	O8—N4—C28—C29	−13.5 (4)
O3—N2—C13—C12	−7.3 (5)	O7—N4—C28—C29	165.2 (3)
O4—N2—C13—C14	−4.9 (5)	C27—C28—C29—C30	1.1 (5)
O3—N2—C13—C14	174.0 (3)	N4—C28—C29—C30	−177.5 (3)
C12—C13—C14—C15	0.3 (5)	C28—C29—C30—C25	−2.4 (5)
N2—C13—C14—C15	179.0 (3)	C26—C25—C30—C29	2.0 (4)
C11—C10—C15—C14	0.1 (4)	C24—C25—C30—C29	−175.3 (3)
C9—C10—C15—C14	173.8 (2)	O1—C1—C31A—C32A	−44.4 (6)
C13—C14—C15—C10	0.2 (4)	C2—C1—C31A—C32A	78.1 (6)
C18—O5—C16—C17	31.9 (3)	S—C1—C31A—C32A	−162.5 (5)
C18—O5—C16—C2	148.64 (19)	C1—C31A—C32A—C33A	174.9 (4)
N1—C2—C16—O5	152.39 (17)	C31A—C32A—C33A—C34A	−178.8 (7)
C1—C2—C16—O5	−85.5 (2)		

### Hydrogen-bond geometry (Å, º)

**Table:**

*D*—H···*A*	*D*—H	H···*A*	*D*···*A*	*D*—H···*A*
C2—H2···O2^i^	0.98	2.55	3.491 (3)	162
C14—H14···O3^ii^	0.93	2.41	3.298 (4)	160

Symmetry codes: (i) −*x*+1, −*y*+1, −*z*+1; (ii) −*x*+1, *y*+1/2, −*z*+3/2.
